# A Functional Precision Medicine Pipeline Combines Comparative Transcriptomics and Tumor Organoid Modeling to Identify Bespoke Treatment Strategies for Glioblastoma

**DOI:** 10.3390/cells10123400

**Published:** 2021-12-02

**Authors:** Megan R. Reed, A. Geoffrey Lyle, Annick De Loose, Leena Maddukuri, Katrina Learned, Holly C. Beale, Ellen T. Kephart, Allison Cheney, Anouk van den Bout, Madison P. Lee, Kelsey N. Hundley, Ashley M. Smith, Teresa M. DesRochers, Cecile Rose T. Vibat, Murat Gokden, Sofie Salama, Christopher P. Wardell, Robert L. Eoff, Olena M. Vaske, Analiz Rodriguez

**Affiliations:** 1 Department of Biochemistry, College of Medicine, University of Arkansas for Medical Sciences, Little Rock, AR 72205, USA; mrreed@uams.edu (M.R.R.); lmaddukuri@uams.edu (L.M.); rleoff@uams.edu (R.L.E.); 2 Department of Neurosurgery, College of Medicine, University of Arkansas for Medical Sciences, Little Rock, AR 72205, USA; adeloose@uams.edu (A.D.L.); mplee@uams.edu (M.P.L.); knhundley@uams.edu (K.N.H.); 3 Department of Molecular, Cell and Developmental Biology, University of California Santa Cruz, Santa Cruz, CA 95064, USA; aglyle@ucsc.edu (A.G.L.); hcbeale@ucsc.edu (H.C.B.); archeney@ucsc.edu (A.C.); anvanden@ucsc.edu (A.v.d.B.); ssalama@ucsc.edu (S.S.); olena@ucsc.edu (O.M.V.); 4 UC Santa Cruz Genomics Institute, University of California Santa Cruz, Santa Cruz, CA 95064, USA; klearned@ucsc.edu (K.L.); ekephart@ucsc.edu (E.T.K.); 5 KIYATEC Inc., Greenville, SC 29605, USA; ashley.smith@kiyatec.com (A.M.S.); tessa.derochers@kiyatec.com (T.M.D.); cecilerose.vibat@kiyatec.com (C.R.T.V.); 6 Department of Pathology, College of Medicine, University of Arkansas for Medical Sciences, Little Rock, AR 72205, USA; gokdenmurat@uams.edu; 7 Howard Hughes Medical Institute, University of California Santa Cruz, Santa Cruz, CA 95064, USA; 8 Department of Biomedical Informatics, College of Medicine, University of Arkansas for Medical Sciences, Little Rock, AR 72205, USA; cpwardell@uams.edu

**Keywords:** Li Fraumeni, glioblastoma, precision medicine, organoid, transcriptomics

## Abstract

Li Fraumeni syndrome (LFS) is a hereditary cancer predisposition syndrome caused by germline mutations in TP53. TP53 is the most common mutated gene in human cancer, occurring in 30–50% of glioblastomas (GBM). Here, we highlight a precision medicine platform to identify potential targets for a GBM patient with LFS. We used a comparative transcriptomics approach to identify genes that are uniquely overexpressed in the LFS GBM patient relative to a cancer compendium of 12,747 tumor RNA sequencing data sets, including 200 GBMs. STAT1 and STAT2 were identified as being significantly overexpressed in the LFS patient, indicating ruxolitinib, a Janus kinase 1 and 2 inhibitors, as a potential therapy. The LFS patient had the highest level of STAT1 and STAT2 expression in an institutional high-grade glioma cohort of 45 patients, further supporting the cancer compendium results. To empirically validate the comparative transcriptomics pipeline, we used a combination of adherent and organoid cell culture techniques, including ex vivo patient-derived organoids (PDOs) from four patient-derived cell lines, including the LFS patient. STAT1 and STAT2 expression levels in the four patient-derived cells correlated with levels identified in the respective parent tumors. In both adherent and organoid cultures, cells from the LFS patient were among the most sensitive to ruxolitinib compared to patient-derived cells with lower STAT1 and STAT2 expression levels. A spheroid-based drug screening assay (3D-PREDICT) was performed and used to identify further therapeutic targets. Two targeted therapies were selected for the patient of interest and resulted in radiographic disease stability. This manuscript supports the use of comparative transcriptomics to identify personalized therapeutic targets in a functional precision medicine platform for malignant brain tumors.

## 1. Introduction

Glioblastoma multiforme (GBM) is the most common malignant primary brain tumor among adults and carries a grim prognosis with less than a 5% chance of 5-year survival following standard therapy. Standard therapy is comprised of surgery followed by radiation and chemotherapeutic temozolomide (TMZ), and this treatment has not changed in 16 years [1,2]. A contributing factor to poor patient response is the one-size-fits-all treatment approach to a heterogeneous tumor, which, despite extensive efforts to genetically subtype, is molecularly unique from patient to patient [3]. This suggests a clear need for the development and identification of personalized treatment strategies, especially for the rare forms of GBM, which result from cancer predisposition syndromes.

Li Fraumeni syndrome (LFS) is a heritable cancer predisposition syndrome characterized by the presence of germline TP53 mutations [4]. TP53 is a tumor suppressor gene activated by stress and/or DNA damage to increase cell cycle arrest, apoptosis, DNA repair, and cell senescence [5,6]. TP53 is the most common mutated gene in cancer and occurs in 30–50% of GBM patients [6,7]. In GBM, mutant TP53 is associated with shorter overall survival and chemoresistance [8,9,10]. Therefore, patients with LFS can serve as an important genetic model to target TP53 mutant tumors.

Genomic profiling of tumors is becoming more frequent and can be used to identify actionable DNA targets for personalized medicine. However, the recent results of the National Cancer Institute’s Molecular Analysis for Therapy Choice (MATCH), a precision medicine trial based on DNA sequencing, demonstrated in a cohort of 4687 patients that only 17.8% of patients qualified to be assigned to therapy [11]. In a cohort of 500 cancer patients, genomic DNA profiling was able to identify potential targets for 29.6% of patients. This percentage increased to 43.4% with the integration of RNA sequencing and immune biomarkers [12]. Tumor RNA sequencing can be used to identify genes and pathways that are significantly expressed in particular patient tumors. However, this analysis is technically challenging to perform for individual tumor samples, especially those which do not have matched normal tissue available for comparison. To address this challenge, Vaske et al. developed a comparative gene expression approach to identify significantly overexpressed genes and pathways of therapeutic significance in individual tumors via comparison to a large compendium of solid tumors [13].

In this manuscript, we combine comparative transcriptomics and ex vivo patient-derived three-dimensional (3D) model systems to identify and validate therapeutic targets. We demonstrate that this pipeline can be used to develop a prospective functional precision medicine platform and show the successful response of the patient of interest to the selected therapies.

## 2. Materials and Methods

### 2.1. UAMS Clinical Cohort

All high-grade glioma (grade III or IV as confirmed by a board-certified neuropathologist based on 2016 World Health Organization (WHO) diagnostic criteria) adult patients who underwent surgical resection in 2018–2019 and had undergone next-generation sequencing were included. Next-generation sequencing included a 595 targeted DNA panel and transcriptome. Within that cohort, our patient of interest was identified as a GBM patient with LFS as confirmed by germline sequencing. The study was approved by the Institutional Review Board.

The LFS patient in this work was also eventually enrolled in 3D-PREDICT, an observational clinical study (ClinicalTrials.gov, accessed on 30 November 2021, identifier NCT03561207) with central and institution-specific IRB approval.

### 2.2. RNA Extraction and Library Construction

RNA sequencing was performed on patients’ tumors using the xT Laboratory Developed Test at Tempus’ Clinical Laboratory Improvement Amendments/College of American Pathologists-accredited laboratory in Chicago, IL. Tumor RNA was extracted from tumor tissue sections with tumor cellularity higher than 20%. Total nucleic acid extraction was performed with a Chemagic360 instrument using a source-specific magnetic bead protocol. RNA was further purified by digestion and magnetic bead purification. The nucleic acid was quantified by Quant-iT Ribogreen RNA Kit (Life Technologies, Carlsbad, CA, USA), and quality was confirmed using a LabChip RNA High HT Pico Sensitivity Reagent Kit (PerkinElmer, Billerica, MA, USA). One hundred nanograms of RNA per tumor sample was fragmented with heat in the presence of magnesium to an average size of 200 base pairs. The RNA then underwent first-strand cDNA synthesis using random primers, followed by combined second strand synthesis and A-tailing, adapter ligation, bead-based cleanup, and library amplification. After library preparation, samples were hybridized with the IDT xGEN Exome Research Panel. Target recovery was performed using streptavidin-coated beads, followed by amplification using the KAPA HiFi Library Amplification Kit. The RNA libraries were sequenced to obtain approximately 65 million reads on an Illumina HiSeq 4000 System using patterned flow cell technology. In parallel, a fresh frozen sample was subjected to polyA RNAseq at Covance, Inc., Wakefield, MA, USA. Briefly, RNA was extracted with the Qiagen RNEasy kit. A sequencing library was prepared with the Illumina TruSeq Stranded mRNA Library Preparation and sequenced on an Illumina HiSeq 2500 sequencer to obtain between 40 and 50 million reads.

### 2.3. Gene Expression Outlier Analysis

All RNAseq data were first uniformly processed using the Toil RNAseq pipeline version 3.2 developed by the UCSC Computational Genomics Lab [14]. Gene-level transcript per million data was used to perform gene expression outlier analysis to identify significantly enriched genes in the patient’s tumor compared with either all 45 grade 3 and grade 4 astrocytoma tumors in the Tempus compendium, 12,747 pediatric and adult tumors in the Tumor Compendium v11 Public PolyA, 912 cell line samples in the Treehouse Cell Line v2 Compendium (available at https://treehousegenomics.soe.ucsc.edu/public-data/#cell_line_v2 accessed 30 November 2021), or tumor types identified as most similar (pan-disease analysis). Separate compendia were used for the Tempus FFPE RNAseq, and polyA fresh frozen RNAseq analysis as these two types of RNAseq data sets are not directly comparable [15]. The Treehouse Cell Line compendium was used for analysis of the cell culture samples since transcriptomic analysis has shown that cell lines can have different molecular profiles from primary tumors or tumor types identified as most similar (pan-disease analysis). For pan-cancer analysis, we used the filtered set of 27,328 genes; for pan-disease analysis, we used the unfiltered set of 58,581 unique GENCODE Human Release 23 genes to make sure we did not miss genes whose expression is specific to certain tumor subtypes.

### 2.4. TumorMap Analysis

The UCSC TumorMap was used to visualize the Treehouse v11 polyA compendium [16]. The method uses a spatial correlation analysis to plot clusters of tumors, which aids researchers in identifying similarities among groups of samples. Each hex in the *TumorMap* plot represents an individual sample. Placement of each hex is determined via pairwise similarity scores between samples causing samples with similar genomic profiles to be plotted near each other and form localized clusters on the *TumorMap*. Spearman rank correlation was used to determine the top six most correlated samples to the patient sample.

### 2.5. DNA Mutation Analysis

Paired normal and tumor samples were processed and sequenced by Tempus to produce Tempus xT targeted panel data. DNA samples were sequenced to a median depth of 1918 (range 436–4643). Reads were aligned to the reference genome GRCh37 using BWA-MEM and variants called using Strelka2 [17,18]. Variants were filtered using FiNGS and annotated using VEP [19,20]. Significantly mutated genes were determined using dNdSCV, with a significance threshold of q < 0.1 [21].

### 2.6. Cell Culture

Primary patient-derived glioblastoma cells (CI0000035099, CI0000034438, CI0000034930, CI0000034840, CI0000035766, CI0000035914) were obtained from the UAMS Tissue Biorepository and Procurement Core within a few hours of surgical removal where tumors were cultured and bio-banked. Consent was obtained from all patients prior to surgery (IRB# 228443). Each specimen was determined by UAMS pathology to be from grade 4 glioblastoma multiforme tumors as per 2016 World Health Organization guidelines. Only early passage cells (<P8) were used for subsequent assays. For clonogenic assays, cells were cultured in minimum essential media (MEM) containing 10% fetal bovine serum (FBS) and 1% (*v*/*v*) antibiotic/antimitotic. For organoid mini-ring assays, cells were cultured in Neurocult NS-A complete media (Stemcell Technologies #05751, Vancouver, BC, Canada) containing NS-A proliferation supplement, 20 ng/mL human recombinant epidermal growth factor (EGF) (Stemcell Technologies #78006), 10 ng/mL human recombinant basic fibroblast growth factor (bFGF) (Stemcell Technologies #78003), 0.0002% heparin (Stemcell Technologies #07980) and 1% (*v*/*v*) antibiotic/antimitotic. Cells were incubated at 37 °C in 5% CO_2_.

### 2.7. RNA Extraction and RNAseq from Cell Cultures

Total RNA was extracted from cell pellets lysed in 600 μL RNA Lysis Buffer using the Quick-RNA Miniprep Kit (Zymo Research, Irvine, CA, USA). Illumina RNA library preparation and sequencing was performed by the Vincent J. Coates Genomics Sequencing Laboratory at UC Berkeley. Total RNA quantity and quality were measured on a NanoDrop One (Thermo Scientific, Waltham, MA, USA) and a Bioanalyzer 2100 (Agilent Technologies, Santa Clara, CA, USA). An input amount of 500 ng high-quality total RNA (RIN score > 8.0 for all samples) was used for library preparation. Libraries were created using the Kapa Biosystems library preparation kit with custom Unique Dual Indexes and analyzed on a Bioanalyzer 2100 (Agilent Technologies). Samples were pooled on a 150PE NovaSeq S4 flowcell and run on a NovaSeq platform with a throughput of 50 M reads per sample.

### 2.8. Clonogenic Assay

From the above-mentioned patient-derived cell lines, 5000 cells per well were plated in a 6-well dish and treated with ruxolitinib (0, 0.1, 1, 10, 100 μM) for 1 h. Media was replaced with drug-free media, and cells were allowed to proliferate for 14 days. Cells were fixed with 3.5% formaldehyde (*v*/*v*) and stained with crystal violet for 30 min at room temperature. Colonies formed were counted using an EVOS FL Auto microscope (Invitrogen, Waltham, MA, USA), where a colony was only counted if it consisted of at least 25 cells. Colonies were graphed as percent survival relative to control-treated cells, where at least 4 biological replicates were plotted per ruxolitinib concentration.

### 2.9. Mini-Ring Organoid Viability Assay

The organoid mini-ring method was adapted from Phan et al., 2019 [22]. Briefly, 8000 cells were combined with hESC qualified Matrigel (Corning, # 354277, Corning, NY, USA) in a 3:4 ratio, and 10 μL of this mixture was plated around the perimeter of each well of a 96-well plate. The Matrigel/cell mixture was solidified at 37 °C for 15 min prior to the addition of Neurocult NS-A complete media. Organoids were grown for three days prior to the addition of 10 concentrations of ruxolitinib (0–100 μM) or everolimus (0–70 µM), 1% DMSO (*v*/*v*). The media was refreshed with fresh drugs every 24 h for a total of 72 h. Organoid mini rings were dissociated into a single-cell suspension via the addition of 50 μL of 5 mg/mL dispase (Life Technologies, #17105-041, Waltham, MA, USA) for 40 min at 37 °C. Viability was determined by the addition of 75 μL/well of CellTiter-Glo 3D Reagent (Promega, #G968B, Madison, WI, USA). Luminescence was measured using the Victor Nivo Multimode Plate Reader (Perkin Elmer, Waltham, MA, USA) at 1 s of integration time per well. Data were normalized to blank wells, and percent viability was calculated relative to vehicle control wells. EC50 values were calculated using Prism 7.

### 2.10. 3D-PREDICT

3D Predict^TM^ Glioma (KIYATEC, Greenville, SC, USA) was performed as described previously [23,24]. Briefly, 3D spheroids were screened for cell survival after exposure to multiple concentrations of single-agent chemotherapies for established periods of time. Calculated IC50 values were translated into response, moderate response, and non-response predictions. A report was provided to the clinician.

## 3. Results

### 3.1. Li Fraumeni Patient Was Identified as a Candidate for Comparative Transcriptomics Pipeline

**Figure 1:**
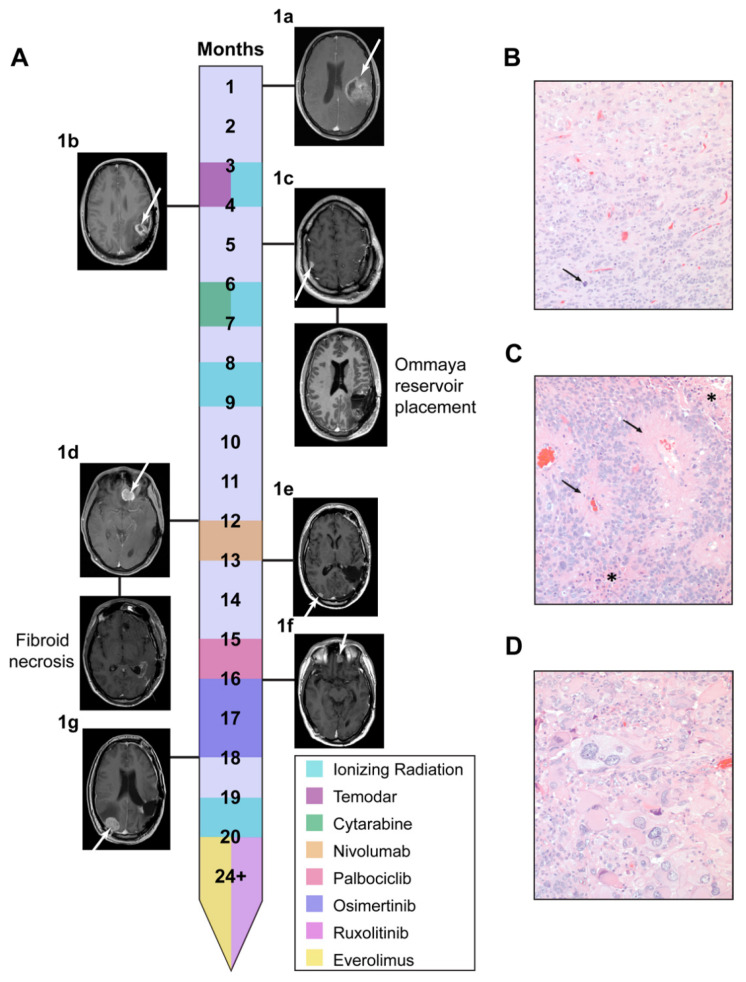
LFS patient was identified as a suitable candidate for comparative transcriptomics. (**A**) Treatment timeline with relevant MRI scans for the patient of interest (Patient 1) is shown. Where the primary tumor is referred to as 1a, and all additional recurrences are labeled 1b-g in order of occurrence. Histopathology findings of the index tumor identified the mass as a high-grade glioma with diffuse (**B**), ependymoma-like (**C**), and pleomorphic xanthrocytoma-like (**D**) area. Mitotic activity (arrow in **B**), necrosis (* in **C**), and pseudorosettes (arrow in **C**) are present at a magnification of 200×.

A 25-year-old male presented with headaches and nausea. He had a history of colon cancer resected at age 3 and a family history for significant cancer in two first-degree relatives. Magnetic resonance imaging (MRI) demonstrated a heterogeneously enhancing 5.4 cm mass in the left temporal lobe (Figure 1A). He underwent a gross total resection, and histopathological analysis of his tumor was notable for glioblastoma with features of ependymoma and pleomorphic xanthoastrocytoma (Figure 1B–D). He underwent standard adjuvant chemoradiation, which ended 8 weeks from diagnosis. He had both local and distal recurrence with the demonstration of a 3 cm lesion adjacent to the previous resection cavity and a 1 cm dural-based lesion overlying the right convexity. These two lesions were resected, and cerebrospinal fluid sampling was positive for malignant cells. He underwent placement of an Ommaya reservoir and underwent intrathecal therapy with the antimetabolite cytarabine. Following completion of his intrathecal therapy, he has had multiple recurrences undergoing further surgery and radiation. Patient 1 was enrolled in 3D-PREDICT 16 months after diagnosis. As such, the last two tumor resections (tumors 1f and 1g) were used in this proprietary functional precision medicine assay. At over 24 months post diagnosis, the patient is still alive with a high functional status.

Molecular markers from the index surgery were identified as follows: IDH wild type, MGMT unmethylated, and EGFR non-amplified. Tumor formalin-fixed paraffin-embedded (FFPE) tissue underwent both DNA and RNA profiling using the xT Laboratory Developed Test, which uses capture technology at Tempus (Chicago, IL, USA). The Tempus xT test revealed a germline mutation in *TP53* p.M40fs, which was classified by Tempus as likely pathogenic. The following somatic mutations were also identified: *TSC2* p.Q1377*, *NF1* p.Y1678fs, and *GPS2* p.Q208*. The *TSC2* and *NF1* mutations were deemed potentially actionable, and Tempus also noted overexpression of MTOR. While the TSC2 mutation in conjunction with high mTOR expression may suggest treatment with everolimus, clinical evidence of activity in glioblastoma has not been supportive [25]. The other aberrations noted by DNA analysis were not suggestive of any treatment options. For this reason, we rationalized that this Li Fraumeni patient may be a suitable candidate for comparative transcriptomic analysis.

### 3.2. Comparative Transcriptomic Analysis Demonstrates Unique Features of the Tumor

**Figure 2:**
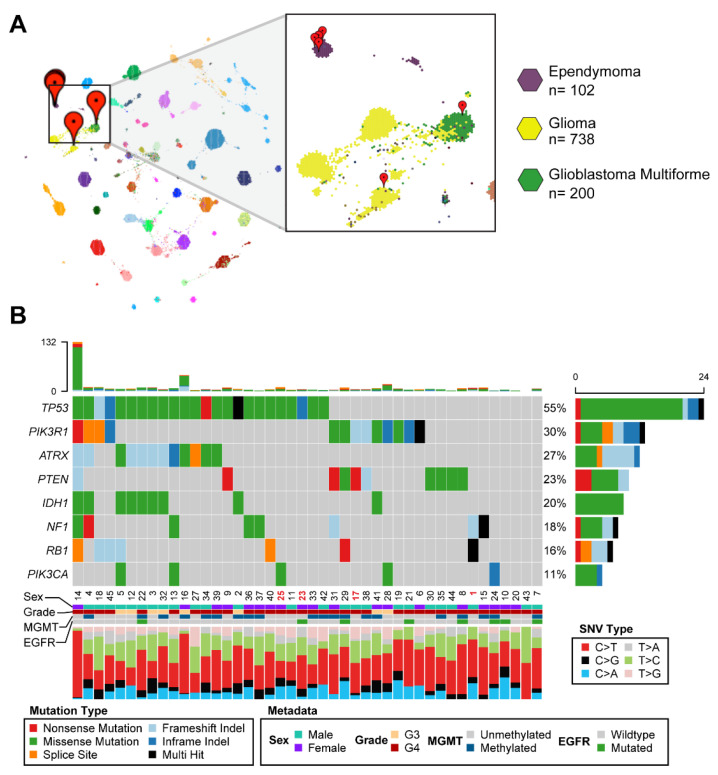
Patient 1 index tumor was extensively characterized using RNA and DNA sequencing. (**A**) TumorMap of Patient 1 relative to the pan-cancer polyA compendium of solid tumors. Spearman correlation was used to identify the top six most correlated samples, which are highlighted with red markers and correlated to tumor type of either ependymoma (purple, 4 tumors), DIPG astrocytoma (yellow, 1 tumor), and glioblastoma (green, 1 tumor). (**B**) Oncoplot depicting somatic mutations present in the UAMS GBM compendium. Mutations were detected using targeted exome sequencing. Barplot at the top shows total number of mutations per sample. Barplot at the right shows number of affected samples. The stacked barplot at the bottom identifies the proportion of each type of single nucleotide variant present. Sex, tumor grade, MGMT methylation status, and EGFR mutation status (assayed via PCR) are also shown below. All 8 genes displayed were significantly mutated in these samples (*p* < 0.1).

Given the histopathological findings in this tumor, we sought to identify tumors that were most similar to the patient’s at the molecular level. We performed TumorMap analysis on the RNAseq data from the fresh frozen tumor sample, comparing it to the data from 12,747 pediatric and adult tumors in the Treehouse v11 polyA compendium (available at https://treehousegenomics.soe.ucsc.edu/public-data/#tumor_v11_polyA accessed 30 November 2021). The expression profile of our patient of interest was highly correlated to various brain tumors. Of the top six most highly correlated samples, four had a diagnosis of ependymoma, one had a diagnosis of GBM, and one had a diagnosis of high-grade diffuse intrinsic pontine glioma (DIPG) (Figure 2A, Table 1). These findings support the initial histopathological report of glioblastoma with ependymoma features. However, it is important to note that no tumor in the pan-cancer compendium, which includes 200 glioblastoma patients, had >95% correlation with our patient of interest, further supporting the idea that traditional treatment would not be beneficial for this patient.

To increase the spectrum of candidate actionable molecular aberrations, we sought to comprehensively identify oncogenes uniquely overexpressed in this patient’s index tumor. Oncoplot analysis of somatic mutations present in the UAMS glioma compendium consisting of 44 other high-grade glioma samples identified additional mutations aside from the germline TP53 mutation in our patient of interest (identified here as Patient 1) (Figure 2B). Multiple mutations in the tumor suppressor RB1 were found along with a frameshift mutation in NF1 (Figure 2B). Mutations in NF1 have also been associated with the development of a central nervous system-centric cancer predisposition syndrome [26]. The somatic mutations in several tumor suppressors, as well as the previously described germline TP53 mutation, likely contributed to the development of GBM in this patient.

### 3.3. Comparative Transcriptomic Analysis Demonstrates Outlier Gene Expression in STAT1 and STAT2

**Figure 3:**
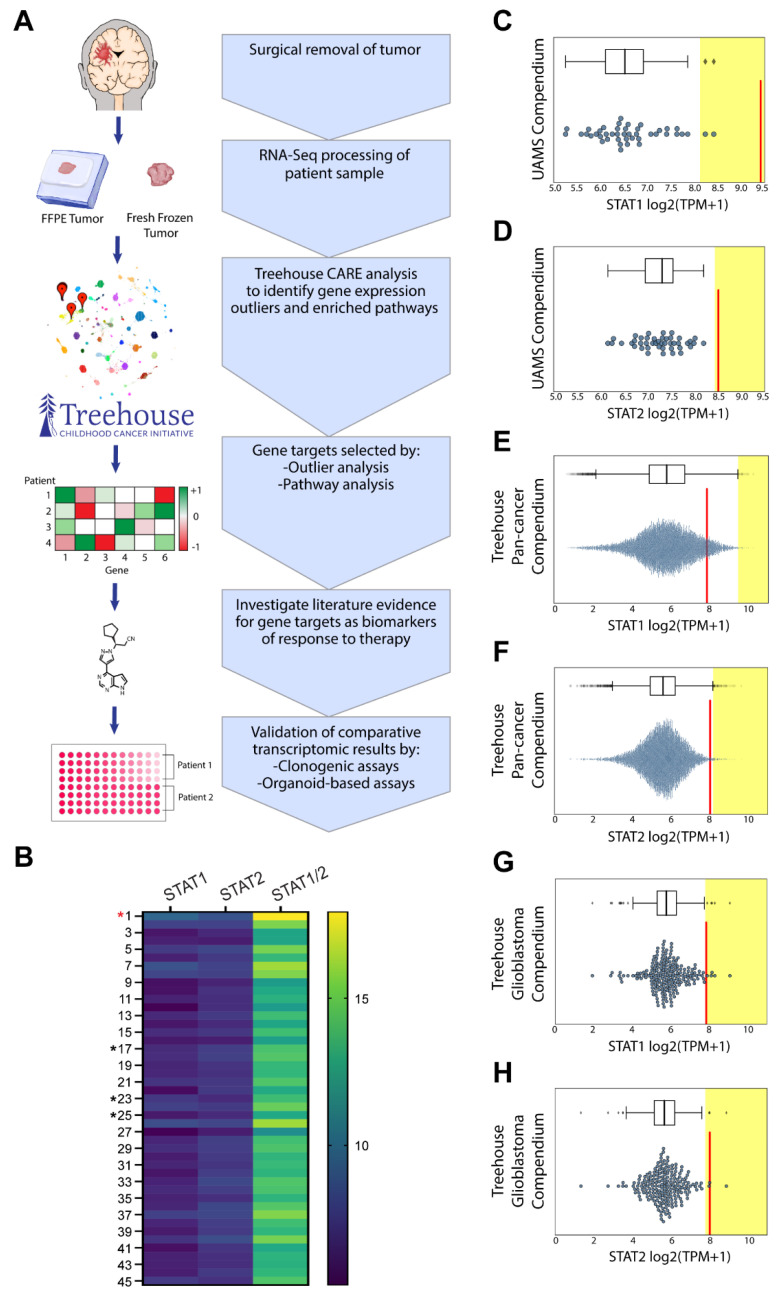
Comparative transcriptomic analysis of Li Fraumeni glioblastoma elucidates novel tumor characteristics. (**A**) Comparative transcriptomic ex vivo functional validation pipeline is described. (**B**) Heatmap identifying *STAT1* and *STAT2* expression from 45 grade III and grade VI astrocytoma patients from the UAMS compendium. Where * identifies patient samples selected for future validation studies and the red * indicates the patient of interest tumor. Patient 1 was compared to the UAMS comparative transcriptomics compendium and identified as having significantly overexpressed *STAT1* (**C**) and *STAT2* (**D**). Where red line indicates patient of interest, and each dot represents a patient tumor sample. Patient 1 was compared to the UCSC pan-cancer tumor compendium consisting of 12,747 tumors, and *STAT1* (**E**) and *STAT2* (**F**) expression levels were identified. Patient of interest RNAseq data were compared to the UCSC glioblastoma compendium consisting of 200 tumors, and *STAT1* (**G**) and *STAT2* (**H**) expression levels were identified.

We then used data generated by both Tempus from the FFPE samples as well as data generated by Covance from the fresh frozen samples to further tease out potential personalized treatment options. We ran the Patient 1 tumor samples through our comparative transcriptomic screening pipeline (Figure 3A). We first compared the expression profile of the FFPE sample to those of 45 other high-grade glioma patients from our UAMS compendium. This analysis demonstrated outlier gene expression in STAT1 (9.4 log2(TPM+1)) and STAT2 (8.5 log2(TPM+1)) in our patient of interest (Figure 3B–D). We then performed gene expression outlier analysis comparing the expression profile of the fresh frozen sample to the expression profiles of 12,747 pediatric and adult tumors in the Treehouse v11 polyA compendia (available at http://treehousegenomics.soe.ucsc.edu/public-data/ accessed 30 November 2021). STAT1 and STAT2 expression was above the median but was not an outlier (Figure 3E,F). This compendium contains data from 1,234 brain tumors, of which 938 are gliomas (grade I–IV). Given that our patient had GBM, gene overexpression was confirmed against a cohort of 200 GBM STAT1 (7.8 log2(TPM+1)) and STAT2 (8.0 log2(TPM+1)) (Figure 3G,H). As gene overexpression was confirmed using two compendia consisting of two separate methods of RNA sequencing, we felt confident in the rigor of our analysis and preceded to a selection of FDA-approved drugs for functional validation studies.

### 3.4. Comparative Transcriptomic Analysis Was Functionally Validated Using Patient-Derived Cell Lines and Organoids

**Figure 4:**
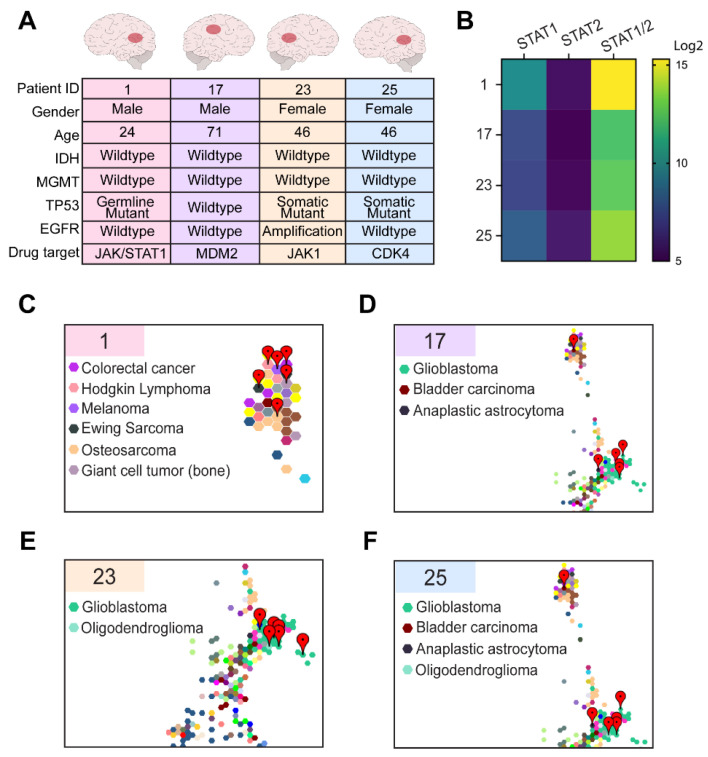
Selected patient-derived cell cultures were characterized prior to functional validation. (**A**) Table identifying tumor location, sex, age, mutation status, and predicted drug targets for the four selected patient cell lines. (**B**) Heatmap identifying *STAT1* and *STAT2* expression for each of the four patient cell lines with combined value shown. TumorMap analysis of selected patient cell lines; Patient 1 (**C**), Patient 17 (**D**), Patient 23 (**E**), and Patient 25 (**F**) with red markers highlighting the top six most correlated cell lines. Colors are correlated with tumor types, as shown next to each TumorMap.

Based on the results from our comparative transcriptomic analysis, we hypothesized that ruxolitnib, a JAK1/2 inhibitor, could be a potential efficacious therapy for indirectly targeting the overexpression of STAT1/2 observed in Patient 1. We sought to validate drug sensitivity using both two- and three-dimensional culture methods. We selected tissue-derived cells from our patient of interest (shown in prior figures as Patient 1), as well as three additional GBM patients (identified as Patient 17, 23, and 25, respectively) from our institutional cohort. These patients were comprised of both genders, spanning from 24 to 71 years old and containing a mix of somatic and germline TP53 mutations (Figure 4A). Cell line RNAseq was used to validate the conservation of gene overexpression in Patient 1. While STAT2 expression was found to be diminished among all cultured patient samples, STAT1 overexpression was maintained in Patient 1 cell culture, and similar trends of STAT1 expression were observed for the other selected tumor lines (Figure 4B). RNAseq data from each of the patient-derived cell lines underwent TumorMap comparison using the Treehouse tumor cell line compendium. The Patient 1 cell line was found to be associated with the most diverse spectrum of cancers relative to the other samples, further supporting the idea that this patient would likely benefit from personalized treatment options (Figure 4C–F, Table 2). For these reasons, we felt confident to proceed with functional validation.

**Figure 5:**
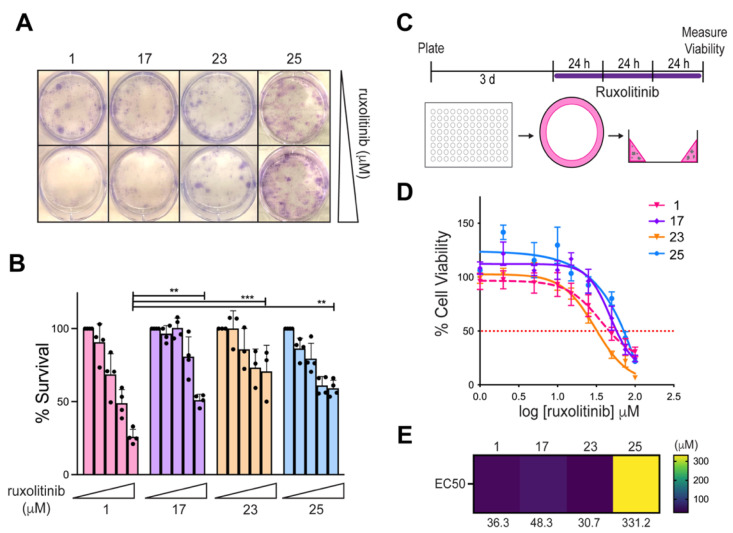
Predicted sensitivity to ruxolitinib was functionally validated. (**A**) Representative clonogenic assay images for 0 and 100 μM ruxolitinib treatment. (**B**) Graph of percent survival of colonies after treatment with ruxolitinib at each of the assayed drug concentrations. Where a colony was only scored if it consisted of at least 25 cells. At least three biological replicates were performed for each cell line, and a significant difference in percent survival between cell lines was determined for 100 μM ruxolitinib treatment using a one-way ANOVA, where ** is *p* ≤ 0.01 and *** is *p* ≤ 0.001. (**C**) Treatment scheme for the patient-derived organoid screening method used. (**D**) Dose-response graphs of the PDO panel. Percent viability was plotted over the log of ruxolitinib concentration to generate EC50 values for each cell line (shown in (**E**)). The red dotted line indicates 50% viability. Each patient-derived organoid drug screen was performed in at least three biological replicates.

A clonogenic assay was first used to examine ruxolitinib sensitivity among our patient cell panel. Cells were treated with increasing concentrations of ruxolitinib (0–100 μM) for one hour, and changes in proliferative potential were measured after 14 days (Figure 5A). Tumor cells obtained from Patient 1 were more sensitive to ruxolitinib than cells from Patients 17, 23, and 25, as evidenced by reduced clonogenic survival (Figure 5B). This effect was most pronounced when comparing clonogenic survival for cells from Patient 1 to the other patient cells treated with 100 μM ruxolitinib (Figure 5B). These survival results were suggestive of increased sensitivity of our patient of interest to the JAK inhibitor; however, we decided to further validate the clonogenic assay with the use of patient-derived organoid (PDO) modeling.

Three-dimensional tumoroid or PDO cultures have been widely adopted in the past few years and have been found to more accurately recapitulate the patient-specific tumor microenvironment [22,27]. Thus, we sought to confirm findings from the clonogenic assay in a more physiologically relevant ex vivo model. Each patient-derived cell line was screened for ruxolitinib sensitivity using a previously described organoid-based viability assay [22]. Patient cells were embedded into Matrigel along the perimeter of each well of a 96-well dish and allowed to grow under three-dimensional culture for three days prior to the start of the assay. PDOs were treated with fresh media containing increasing concentrations of ruxolitinib every 24 h prior to viability measurement at 72 h (Figure 5C). Interestingly, Patient 1 was found to have similar drug sensitivity to another patient sample, Patient 23 (Figure 5D,E). Using comparative transcriptomics, we found that Patient 23 had overexpression of JAK and was a suitable candidate for ruxolitinib treatment (Figure 4A). In this manner, we confirmed prior reports that comparative transcriptomic analysis is useful for the identification of candidate genes that can be targeted either directly (JAK) or indirectly (STAT1/2) [13]. Additionally, the two patient tumors that were not predicted to be responders to ruxolitinib treatment were found to be the least sensitive to this drug (Figure 5D,E). The sensitivity of these two samples was found to inversely correspond to STAT expression, as Patient 25 with the lowest STAT1/2 expression (12.8 log2(TPM+1)) was found to be the most resistant to ruxolitinib (Figure 5D,E). These results strongly support the predictive power of comparative transcriptomics and our functional validation platform.

### 3.5. 3D-PREDICT Trial Identifies mTOR Inhibition as an Additional Therapeutic Target

**Figure 6:**
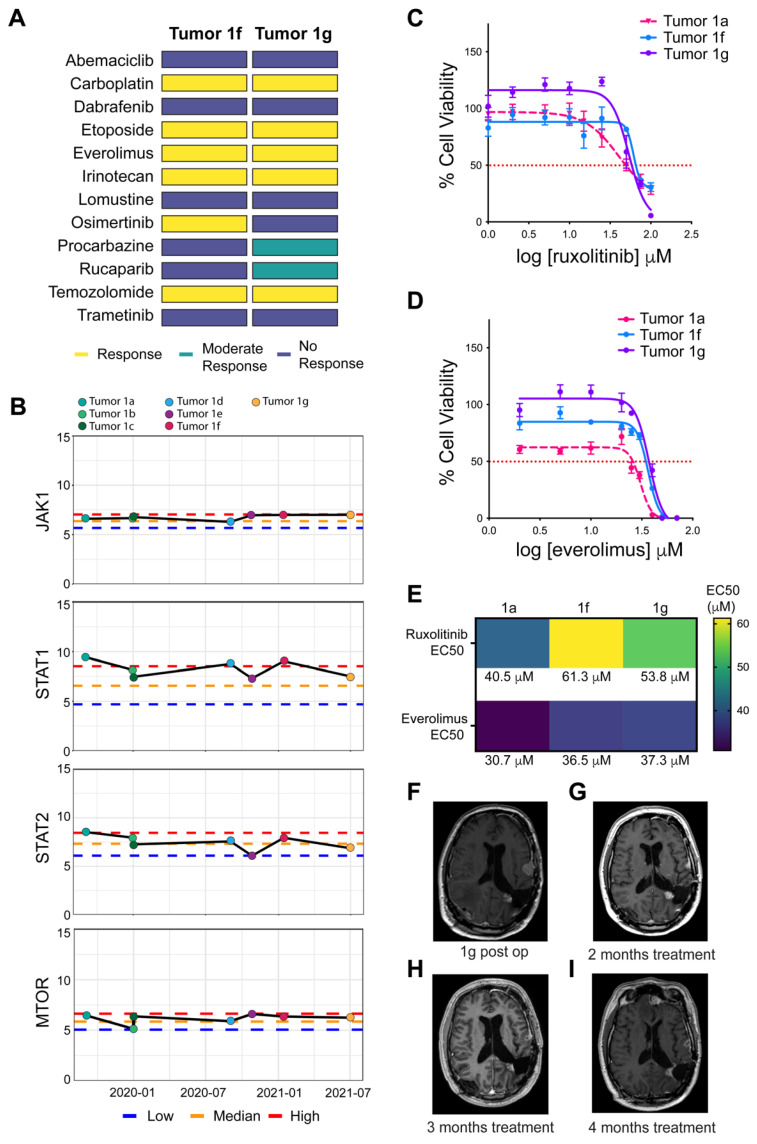
Patient 1 was found to benefit from a combination of JAK/STAT and mTOR inhibition. (**A**) 3D-PREDICT response panels for tumors 1f and 1g. Yellow indicates drug response, teal indicates moderate drug response, and dark blue indicates no drug response. (**B**) Graphs of longitudinal log2(TPM+1) gene expression analysis of JAK1, STAT1, STAT2, and mTOR of all LFS patient tumors 1a-1g (color-coded in key above graphs). Gene expression was recorded relative to the UAMS GBM compendium and was plotted as either low, median, or high expression. Dose-response graphs of index (1a) and recurrent (1f and 1g) patient-derived organoids were generated. Percent viability was plotted over the log of ruxolitinib (**C**) or everolimus (**D**) concentration to generate EC50 values for each tumor. The red dotted line indicates 50% viability. Each patient-derived organoid drug screen was performed in at least three biological replicates. (**E**) Heatmap showing EC50 for all tumors after treatment with either ruxolitinib or everolimus. (**F**) MRI of Patient 1 after resection of tumor 1g. MRI scans were repeated at 2 (**G**), 3 (**H**), and 4+ (**I**) months of concurrent treatment with ruxolitinib and everolimus and were confirmed by radiology to show stable disease in each scan.

Concurrent to the comparative transcriptomic analysis of the LFS patient’s index tumor, this patient was enrolled in the 3D-PREDICT clinical study prior to his sixth recurrence (ClinicalTrials.gov, accessed on 30 November 2021, ID NCT03561207). This observational clinical study protocol has IRB approval to enroll patients with epithelial ovarian cancer, high-grade gliomas, and high-grade rare tumors. Tissue was collected from recurrent tumors 1f and 1g (Figure 1A), 3D spheroids were then generated from dissociated cells from each tumor and treated with a panel of 12 drugs at multiple concentrations. Cell viability measurements were used to elucidate tumor-specific responses for each of these 12 compounds (Figure 6A).

Using this spheroid-based screening assay, tumor 1f was found to be responsive to 6 of the 12 compounds. An oncologist reviewed the panel of responding drugs with Patient 1, and based on the risk profile and previously recorded patient outcomes in the literature, the EGFR inhibitor osimertinib was selected for therapeutic use. Recurrence occurred within two months of treatment (Figure 1A). Spheroid testing on the 3D-PREDICT study was performed on recurrent tumor 1g, and this subsequent tumor was found to be resistant to osimertinib (Figure 6A). Therapeutic elimination of osimertinib-sensitive clones within tumor 1f, coupled with the aggressiveness of other malignant clones, may factor into disease progression. The positive correlation of changes in tissue response of these serial specimens is consistent with the clinical response observed in this patient. Further supporting the prediction of the clinical response is tumors 1f and 1g tested on the 3D-PREDICT study had no response to abemaciclib, a CDK4/6 inhibitor similar to palbociclib. This finding is consistent reported disease progression of Patient 1 while on palbociclib, seen on the treatment timeline (Figure 1A) prior to tumors 1f and 1g. Positive correlations between serial tissue responses versus therapeutic response have been documented in other patients with high-grade glioma tested with this 3D spheroid platform as well [24]. One of the compounds found to which both tumors 1f and 1g were responsive was the mTOR inhibitor everolimus. This was promising as the index tumor (1a) was previously found by Tempus to have an overexpression of mTOR, supporting everolimus as a potential therapeutic target.

We performed comparative transcriptomics analysis on all seven tumors from Patient 1 to further dissect how treatment-selective pressure had shaped the tumor microenvironment. We examined gene expression levels of both JAK/STAT as well as mTOR in this tumor panel (Figure 6B). These gene comparisons were made via our UAMS GBM cohort and suggest that the final two tumors (1f and 1g) have sustained high levels of JAK1, above median expression of STAT1 and median expression of STAT2. Additionally, while not a significant outlier, mTOR expression was found to be consistently above the median expression level for both the index tumor as well as tumors 1f and 1g (Figure 6B).

We further validated drug response to everolimus and ruxolitinib on the index tumor 1a as well as recurrent tumors 1f and 1g (Figure 6C,D). The index tumor was found to be the most responsive to ruxolitinib, which corresponds to the high levels of STAT1/2 recorded in this tumor. There was only a slight (~13–20 μM) drop in sensitivity to ruxolitinib in cells from 1f and 1g, which corresponded to decreased STAT1/2 (Figure 6B). For all three tumors, mTOR expression was reported as being comparably high, and this seemed to correspond with consistent sensitivity to everolimus (Figure 6D,E). From these results, Patient 1 elected to try a combination therapy consisting of ruxolitinb and everolimus, and MRI results from 2 and 3 months on treatment show stable disease (Figure 6F–H). Additionally, as of >4 months on this treatment, the patient remains classified as having stable disease (Figure 6I).

## 4. Discussion

In this study, we demonstrate the feasibility of using comparative transcriptomics analysis to identify personalized drug treatments. These potential therapies were then functionally validated using patient-derived organoid screening methods. Our patient of interest was a young man with LFS, an autosomal dominant cancer predisposition syndrome caused by a germline mutation in *TP53* [4,27,28,29]. LFS leads to an increased risk in the development of many cancers, inclusive of breast cancer, sarcoma, lymphoblastic leukemia, adrenocortical carcinoma, and brain tumors. The three most commonly associated brain tumors with LFS are choroid plexus papilloma, medulloblastoma, and glioma [30]. Gliomas in patients with LFS have not been extensively studied, but the genomic characterization of 14 patients demonstrates clinical stratification into two groups based on *IDH* mutation status. Patients with *IDH* wildtype (*IDH*wt) status have a worse prognosis and younger presentation with a median age of diagnosis of 6 years [31]. As our patient is *IDH*wt but presented as a young adult, this makes him the oldest reported LFS patient with *IDH*wt high-grade glioma in the literature to date. His tumor had unique histopathological characteristics, which demonstrated features of ependymoma and pleomorphic xanthoastrocytoma (Figure 1B–D). These findings correlated with our comparative gene expression analysis using TumorMap, which demonstrated the highest tumor similarity to four ependymoma cases as well as one GBM and one DIPG (Figure 2A). No tumor in our large cancer compendium of 12,747 samples had >95% correlation with our patient of interest, demonstrating the unique gene expression characteristics of our clinical case and the potential that standard therapy may not be beneficial.

Identification of actionable targets using genomics has been the basis of many therapeutic trials in cancer. The NCI-MATCH trial used next-generation sequencing to identify therapeutically actionable molecular alterations and demonstrated the feasibility of this paradigm in a large cohort [32]. Only <20% of the population qualified for targeted therapy [11]. However, certain targeted therapy such as AKT inhibitors for patients with AKT1 mutated metastatic tumor demonstrated objective clinical response using a single agent [33]. These results are promising, and more post-hoc analyses of the NCI-MATCH data are ongoing. Integration of transcriptome data can further increase the number of identified targets for a patient’s tumor as many patients do not have actionable DNA alterations [12]. This is especially true for tumors of younger patients, which tend to harbor fewer DNA mutations compared to tumors in older adults.

In this manuscript, we performed both DNA and RNA analysis on our patient. In addition to the germline mutation in TP53, the DNA analysis revealed somatic stopgain mutations in the TSC2 and NF1 tumor suppressor genes, as well as a stopgain mutation in GPS2 (Figure 2B). None of these variants could be targeted clinically, and so we used the comparative transcriptomics workflow developed by Vaske et al. to identify additional molecular aberrations that could be therapeutically targeted (Figure 3A) [13]. The comparative transcriptomics workflow places the individual tumor profile in the context of a larger cohort of cancer and normal tissues allowing for the identification of tumors that are most similar to the given tumor and detection of transcripts uniquely overexpressed in the given tumor sample. For this case, we performed comparative analysis twice, using both an FFPE and fresh frozen sample from the index tumor. The FFPE sample was compared against a small compendium of RNAseq data generated from 45 glioma samples at our institution in the 2018–2019 year (available at https://treehousegenomics.soe.ucsc.edu/public-data/#tumor_21.02_hybridcapture accessed 30 November 2021) (Figure 3C–H). Our comparative transcriptomic analyses recapitulated the pathology findings of ependymoma elements in this tumor. In addition, both the FFPE and fresh frozen tumor analysis revealed overexpression of *STAT1* and *STAT2*, even though two different platforms and two separate reference compendia have been used in the analyses.

To validate our STAT1/2 gene expression outlier findings, we used a tumor organoid model (Figure 5). Patient-derived xenografts (PDX) are frequently used to recapitulate the characteristics of the parent tumor [34]. However, a main disadvantage of PDXs includes the length of time required to grow the tumor, making the feasibility of use in real-time clinical precision medicine decisions less likely. Three-dimensional tumor cultures, such as organoids and spheroids, have been shown to more closely recapitulate the patient tumor microenvironment in several types of solid tumors relative to more traditional adherent culture. Multiple GBM patient-derived 3D models now exist to recreate the tumor microenvironment [22,27,35]. In our study, we used a colony formation assay as well as adapted a medium-throughput organoid mini-ring method to generate EC50 values from patient-derived tumor cells within a week [22]. For various cancer types, inclusive of GBM, PDO models have been used for drug screening [23,36].

Our viability and EC50 data correlated with the STAT1 expression level of the parent tumor; however, the knowledge of the downstream signaling pathway(s) that certain TP53 mutations mediate JAK1/STAT1 overexpression and glioma-genesis is unknown. STAT1, a protein involved in interferon (IFN) signal transduction, is known to mediate both tumor-suppressive and tumor-promoting pathways [37]. In GBM, STAT1 promotes tumorigenicity, and increased expression correlates with poor patient survival [38]. Aberrant STAT1 activation triggers radioresistant signaling pathways in head and neck, renal, breast, and myeloma cancer [39]. STAT1 has also been shown to become activated by double-strand break (DSB) formation, which has implications in treatment response with standard of care DNA damaging agents (i.e., temozolomide, a DNA alkylating agent, and radiation), repair of which are mediated by upregulation of the DNA damage response (DDR) [40,41,42,43]. Therefore, targeting DDR through pathways such as STAT1 could potentially serve as an adjuvant therapy in GBM.

Recurrent tumors from Patient 1 were screened using the 3D-PREDICT spheroid assay to examine sensitivity to a panel of 12 compounds (Figure 6A). Sensitivity to the mTOR inhibitor everolimus was reported through this method in both recurrent tumors 1f and 1g. Interestingly, Tempus performed on the index tumor had suggested mTOR as a possible therapeutic target, even though prior clinical trials of everolimus in GBM have shown no significant increase in survival [25]. Further functional studies provided additional evidence to support concurrent treatment with ruxolitinib and everolimus (Figure 6C–E). At the date of this publication, Patient 1 remains on this treatment regime and is classified as having a stable disease.

Our results are promising and demonstrate the feasibility of a functional precision medicine platform that incorporates comparative transcriptomics and patient-derived organoids. We leverage a bioinformatics pipeline to identify potential targetable pathways exclusive of the presence or absence of actionable mutations. Following the bioinformatics pipeline, these identified targets can be used in ex vivo patient-derived models to validate efficacy.

## Figures and Tables

**Table 1:** List of spearman correlation values of the top six highest correlated tumors to that of Patient 1.

Sample ID	Spearman Rank Correlation	Disease
THR24_1804_S01	0.873	Ependymoma
THR24_1821_S01	0.872	Ependymoma
THR13_0969_S01	0.866	Glioma
THR24_1839_S02	0.864	Ependymoma
THR24_1786_S01	0.861	Glioblastoma multiforme

**Table 2:** Spearman rank correlation values of the top six highest correlated cell lines for each of the patients described in Figure 4.

Tumor ID	Sample ID	Spearman Rank Correlation	Disease
	G28816	0.872	Ewing sarcoma
	G28810	0.872	Cutaneous melanoma
Patient 1	G28809	0.869	Colorectal carcinoma
	G28895	0.868	Osteosarcoma
	G28837	0.868	Malignant giant cell tumor of bone
	G28818	0.867	Hodgkin lymphoma
	G27543	0.837	Glioblastoma
	G26232	0.834	Glioblastoma
Patient 17	G26195	0.833	Glioblastoma
	G26217	0.833	Glioblastoma
	G28902	0.832	Bladder carcinoma
	G27255	0.831	Anaplastic astrocytoma
	G26217	0.873	Glioblastoma
	G26232	0.869	Glioblastoma
Patient 23	THR13_0970_S02	0.861	Glioblastoma
	G27205	0.860	Glioblastoma
	G27462	0.858	Glioblastoma
	G26228	0.858	Oligodendroglioma
	G28902	0.846	Bladder carcinoma
	G26217	0.834	Glioblastoma
	G26228	0.833	Oligodendroglioma
Patient 25	G26232	0.831	Glioblastoma
	G27255	0.826	Anaplastic astrocytoma
	G26195	0.826	Glioblastoma

## Data Availability

The raw data for the polyA RNAseq from the patient’s tumor as well as the patient-derived cell lines can be found at EGA (GSE188739). The three gene expression compendia used in this analysis are available at https://treehousegenomics.soe.ucsc.edu/public-data/ accessed 30 November 2021.
